# Late enhancement and washout maps for distinguishing tumor progression from radiation necrosis in malignant brain tumors: an exploratory study

**DOI:** 10.1186/s12885-026-17170-0

**Published:** 2026-10-15

**Authors:** Eya Khadhraoui, Leon Schmidt, Stefan Klebingat, I Erol Sandalcioglu, Roland Schwab, Philipp Arndt, Daniel Behme, Klaus-Peter Stein, Sebastian Johannes Müller

**Affiliations:** 1 Clinic for Neuroradiology, Otto-von-Guericke University Magdeburg, Leipziger Str. 44, D- 39120 Magdeburg, Germany; 2 Department of Neurosurgery, Otto-von-Guericke University Magdeburg, Leipziger Str. 44, D-39120 Magdeburg, Germany; 3 Stimulate Research Campus Magdeburg, Otto-Hahn-Straße. 2, D-39106 Magdeburg, Germany; 4 Clinic for Neurology, Otto-von-Guericke University Magdeburg, Leipziger Str. 44, D-39120 Magdeburg, Germany; 5 Clinic for Neuroradiology, Hannover Medical School, Carl Neuberg Str. 1, D-30625 Hannover, Germany; 6 Department of Neurosurgery, Charité - Universitätsmedizin Berlin, Luisenstr. 64, D-10117 Berlin, Germany

**Keywords:** Late enhancement, Contrast clearance, Washout, MR perfusion, Progression, Radiation necrosis, Glioblastoma

## Abstract

**Background:**

Distinguishing between tumor progression and radiation necrosis during brain tumor follow-up is challenging. Advanced MR imaging techniques, such as perfusion, diffusion-weighted imaging (DWI), and spectroscopy, can help differentiate between these conditions. In our clinic, we established a novel technique, the rapid washout and late-enhancement map. This study investigated the value of this map in distinguishing true progression from radiation necrosis.

**Methods:**

Patients with a histologically confirmed initial diagnosis of glioblastoma, high-grade astrocytoma, primary central nervous system lymphoma, or brain metastasis who were undergoing radiochemotherapy and participated in our MRI protocol, including MR perfusion and rapid washout imaging, between January 1, 2024, and December 31, 2025, were included in the study.

We volumetrically measured late enhancement and washout in brain tumors at all follow-up examinations and analyzed the resulting volumes. Additionally, we evaluated diffusion-weighted imaging and MR perfusion images.

**Results:**

A total of 33 patients (25 with glioblastoma, two with astrocytoma grade 4, two with astrocytoma grade 3, one with CNS lymphoma, and three with brain metastasis) were included. Diagnostic confirmation was obtained by histopathological assessment in 20 patients, MRI follow-up in 13 patients, with additional fluorodeoxyglucose-positron emission tomography in two.

Among these patients, 23 were diagnosed with tumor recurrence, four with mixed pathology comprising “radiation necrosis with scattered active tumor cells”, and six with radiation necrosis. The mean washout ratio (± standard deviation) was 31 ± 18 % for progressive disease, 35 ± 20 % for mixed pathology, and 10 ± 7 % for radiation necrosis, respectively.

**Conclusions:**

The washout ratio can enhance the differentiation between recurrent tumor growth and radiation necrosis during post-treatment monitoring of malignant brain tumors. As MR perfusion can yield inconclusive results in this setting, the proposed method offers complementary information that supports initial diagnostic decision making. Diagnostic discrimination may be strengthened by combining the relative cerebral blood volume with washout ratio analysis, rather than relying on a single imaging marker.

**Supplementary Information:**

The online version contains supplementary material available at https://doi.org/10.1186/s12885-026-17170-0.

## Introduction

Aggressive primary and secondary brain tumors in adults, such as glioblastoma (Gb), high-grade gliomas, central nervous system (CNS) lymphomas, and brain metastases, are associated with poor prognosis despite multimodal treatment approaches, including surgery, radiotherapy, and chemotherapy [1]. Consequently, accurate assessment of treatment response and early detection of tumor progression are crucial for optimal patient management and therapeutic decision-making [2, 3].

MRI, particularly contrast-enhanced T1-weighted imaging, is the cornerstone of response assessment in GBM and forms the basis of the widely used Response Assessment in Neuro-Oncology (RANO) criteria [4]. According to these criteria, changes in contrast enhancement are key indicators of tumor progression [5]. However, the interpretation of post-treatment MRI findings remains challenging because therapy-related changes, such as radiation necrosis and pseudoprogression, can mimic tumor progression by producing similar patterns of contrast enhancement [6].

This overlap substantially limits the specificity of conventional MRI and complicates reliable response assessment [7]. Consequently, distinguishing true tumor progression from post-therapeutic changes remains a central challenge in neuro-oncologic imaging [8]. The most recognized advanced imaging techniques are MR perfusion, MR spectroscopy, and diffusion-weighted imaging [9]. PET (Positron Emission Tomography) imaging is also a possibility [10].

Evidence from experimental research shows that high-grade gliomas tend to demonstrate peak enhancement around 3–8 minutes’ post-contrast injection [11, 12], a temporal characteristic that can be used to enhance MRI-based diagnosis.

Based on these findings, the imaging protocol was optimized by acquiring 3D T1-weighted sequences approximately 5 and 25 min after contrast injection. This temporally resolved acquisition strategy was designed to enhance the differentiation between tumor progression and radiation-induced necrosis, as demonstrated in a feasibility study [13].

In principle, this approach involves the co-registration of two T1-weighted image sets, followed by intensity normalization and voxel-wise subtraction. The derived difference map is then displayed with a red-green color scheme, assigning red to washout effects and green to delayed or late enhancement.

Amino acid PET, particularly ^18^F-fluoroethyl-L-tyrosine PET (FET PET), can support the differentiation of recurrent glioma from treatment-related changes when MRI findings are inconclusive [14]. However, its availability varies because it requires specialized PET infrastructure and access to the radiotracer [15].

**Fig. 1:**
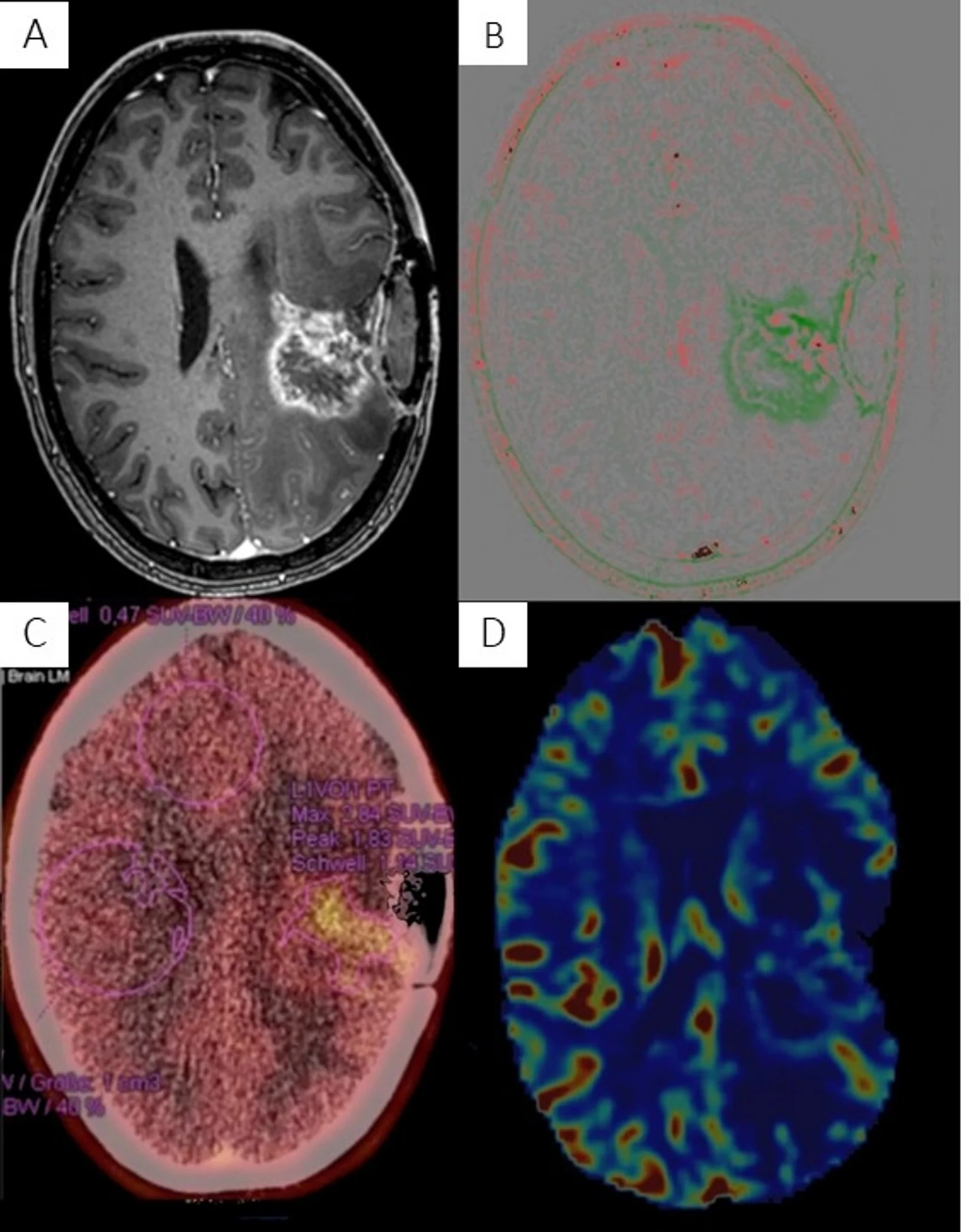
Introduction example. A patient with an initial diagnosis of astrocytoma grade 3 developed a new contrast-enhancing tumor-like lesion, three months after surgery, as demonstrated on axial contrast-enhanced T1-weighted images (**A**). Washout (**B**) and MR Perfusion (**D**) revealed no active tumor cells. Surgery was performed because of the positive FET-PET in the rostral tumor region (**C**) and chronic epidural hematoma with compression of the peritrigonal region. Histological examination revealed radiation necrosis but no active tumor cells

Figure 1 illustrates a representative example of radiation necrosis.

The aim of the current study was to investigate whether this delayed-enhancement approach could also improve the differentiation between true progression and radiation necrosis in the follow-up of malignant brain tumors.

## Methods

### Study design

 Ethical approval for this single-center observational study was granted by the Institutional Review Board, and the study conformed to the principles of the 2013 Declaration of Helsinki. In view of its retrospective nature and anonymized dataset, informed consent was waived. All methods adhered to relevant regulatory standards.

### Participant population

Our Picture Archiving and Communication System (PACS) was queried to identify patients with valid rapid washout protocols between 01/01/2024 and 31/12/2025.

The included diseases were histologically proven glioblastoma, other high-grade gliomas of WHO grade 3–4, CNS lymphoma, or brain metastasis according to the 2021 WHO CNS tumor classification [16].

Patients with new contrast-enhancing lesions on follow-up MRI after surgery and radiochemotherapy were included in this study. This includes suspected progressive disease, radiation necrosis, and pseudoprogression based on MRI findings.

The availability of a complete MRI tumor protocol was an additional inclusion criterion.

Exclusion criteria comprised: (1) histopathological entities other than glioblastoma, WHO grade 3–4 gliomas, CNS lymphoma, or cerebral metastasis; (2) imaging limitations such as motion artifacts or absent contrast enhancement that made wash-out map computation impossible; (3) patients younger than 18 years; (4) no measurable contrast-enhancing volume > 125 mm³ (5 × 5 × 5 mm); and (5) a history of another primary malignancy other than the tumor entity under investigation.

### MRI protocol and technical details

All MRI scans were acquired using Siemens Sola 1.5T (Siemens Healthineers AG, Munich, Germany), Philips Achieva 3T, and Philips Intera 1.5T scanners (Koninklijke Philips NV, Amsterdam, Netherlands). The acquisition protocol and washout software have been previously published [13]. The imaging protocol was standardized, with the first post-contrast T1-weighted sequence acquired 5–9 min after contrast administration and the second T1-weighted sequence acquired 25–29 min after contrast administration. Imaging included DSC perfusion MRI without a pre-bolus (automated leakage-correction provided by the manufacturer-dependent post-processing software) and echo-planar imaging (EPI) diffusion-weighted imaging. Washout maps were generated using a Python script that retrieved both T1-weighted sequences from PACS. These datasets were co-registered using the FMRIB FLIRT algorithm [17–20], then intensity-normalized, and subtracted voxel-wise. The final maps were exported to the PACS. A detailed description of the methodology is provided in the Supplementary Materials.

### Measurements

All assessments were conducted independently by two readers and averaged for subsequent analysis. Interrater reliability was determined using the intraclass correlation coefficient (ICC (2, k)) with a two-way random-effects model and average measures. For each case, total contrast-enhancing volume as well as wash-out and wash-in (delayed enhancement) volumes were segmented using 3D Slicer (level-trace tool), applying a semi-automated slice-by-slice technique. Additional metrics (washout ratio) were derived according to a previously published TRAMs study [21].

The volume of increased relative cerebral blood volume (rCBV) in the contrast-enhancing lesions was also estimated using the 3D Slicer. We chose a clinically established threshold of > 1.5 for these measurements [22].

Additionally, we assessed the volume of significant (> 10%) reductions in apparent diffusion coefficient (ADC) values in contrast-enhancing lesions compared to the healthy hemisphere.

### Reference standard and diagnostic classification

Histopathology was considered the reference standard when tissue was available. In patients without histological confirmation, the final diagnosis was based on longitudinal clinical and MRI follow-up of at least 6 months. Progressive enlargement of the enhancing lesion on serial MRI was considered indicative of tumor recurrence, whereas sustained stability or regression was considered indicative of radiation necrosis. Histopathological specimens showing both radiation necrosis and viable tumor cells were classified as mixed pathology. FET-PET findings were used as supportive diagnostic information, with increased tracer uptake relative to normal cortical grey matter considered suggestive of viable tumor.

The primary statistical unit was the patient. Although 41 lesions were identified in 33 patients, only one lesion per patient was included in the primary analysis to avoid violating the independence assumption. In patients with multiple lesions, lesion selection followed a hierarchical rule: a histopathologically confirmed lesion was selected whenever available; if no lesion had histopathological confirmation, the lesion demonstrating fastest growth during follow-up was selected. Thus, the patient-level analysis included 33 lesions from 33 patients. A lesion-level analysis including all lesions was performed only as a sensitivity analysis.

### Statistical analysis

Statistical analyses were performed using Python 3 (version 3.11, Matplotlib) and R (r-project.org, version 4.5.3). Group comparisons were conducted using Kruskal Wallis tests, with statistical significance set at *p* < 0.05. Group comparisons of categorical frequencies, such as the proportion of patients with elevated rCBV (rCBV > 1.5) across the three diagnostic groups, were performed using Fisher’s exact test for a 3 × 2 contingency table. A two-sided p value < 0.05 was considered statistically significant. 95% confidence intervals (CI) were calculated. The intraclass correlation coefficient ICC(2,k) was computed in Python 3 using the “pandas” and “pingouin” libraries and interpreted according to Koo and Li [23].

### Multivariable analysis

A multivariable logistic regression model (“model <- glm(Type ~ ADC + WO + rCBV, data = data_csv, family = binomial)”) was performed with R (library pROC) to assess the combined diagnostic value of ADC, washout ratio, and perfusion parameters for differentiating radiation necrosis from tumor progression and mixed pathology. The predicted probabilities derived from the model were used for Receiver operating characteristic (ROC) analysis, and the optimal threshold was determined using the Youden index.

## Results

### Participants

**Fig. 2:**
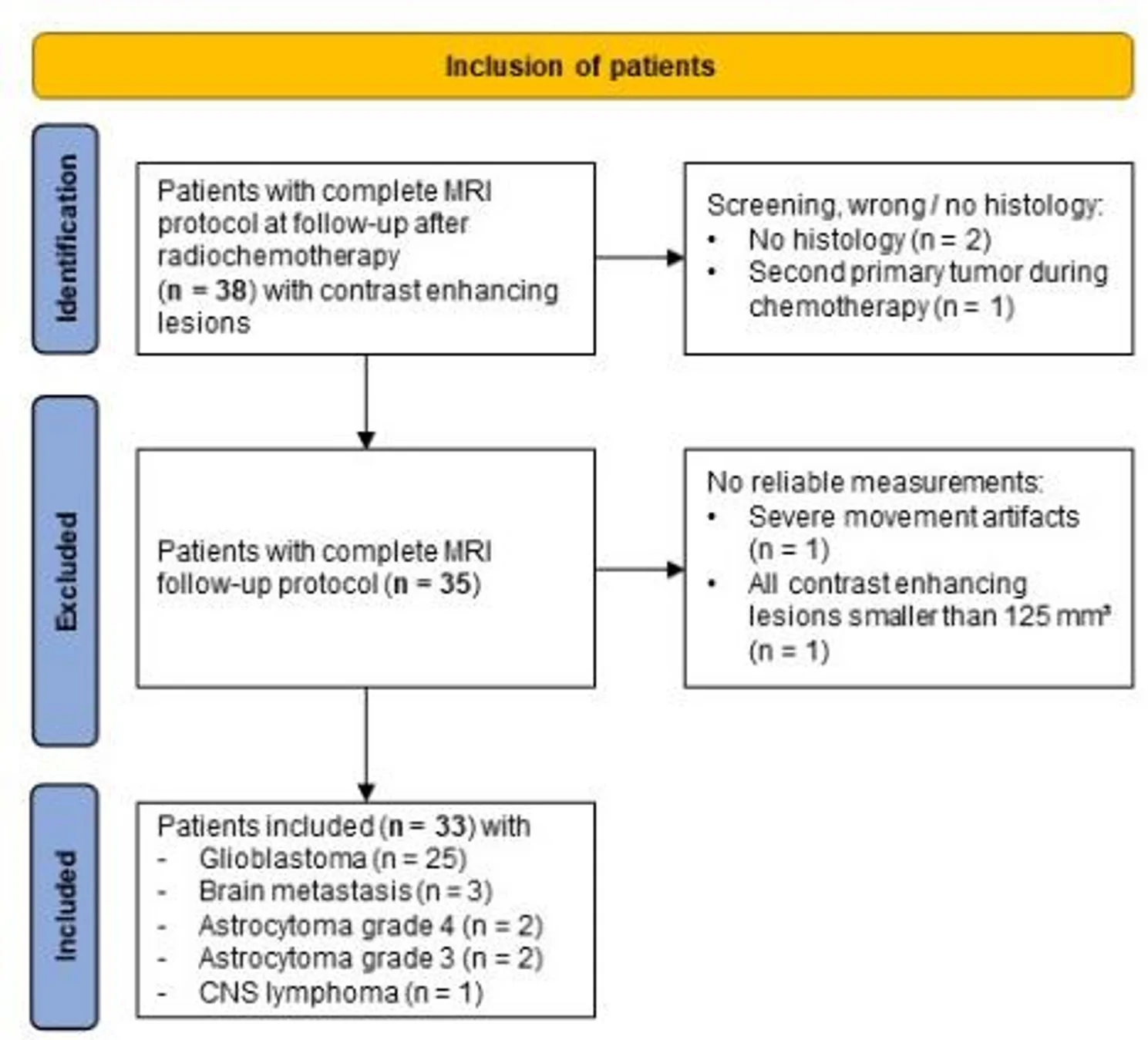
Inclusion flow chart

A total of 38 patients with complete follow-up MRI tumor protocols, including rapid washout maps and contrast-enhancing volumes exceeding 125 mm³, were identified through our PACS search. Three patients were excluded based on histological mismatch, and two patients were excluded due to T1-weighted imaging errors, such as absent enhancement or severe motion artifacts. The final cohort comprised 33 patients (25 with glioblastomas, 4 with astrocytomas, 1 with CNS lymphoma, and 3 with brain metastases). The selection process is shown in Fig. 2.

The mean age of the 33 patients was 58.8 ± 12.2 years (mean ± standard deviation), including 13 women. The detailed statistics are shown in Suppl. Table S1.

The detailed volumes of the measurements are listed in Table 1.

**Table 1:** **–** Mean ± standard deviation of measurements. P-values were calculated using Kruskal Wallis and Fisher’s exact test

Subgroup	Progressive disease (23 patients)	Mixed pathology (4 patients)	Radiation necrosis (6 patients)	*p* value
Contrast enhancing tumor volume (mm³)	8306 ± 11,728	12,435 ± 17,705	14,291 ± 15,739	0.53
rCBV increased lesion volume in patients with MR perfusion	48% 10/21	75% 3/4	17% 1/6	0.25
rCBV increased volume (mm³)	2990 ± 4568 (*n* = 10)	4276 ± 6534 (*n* = 3)	1564 (*n* = 1), **	*
Wash-out volume (mm³)	3486 ± 7399	2626 ± 3602	1076 ± 910	0.49
Wash-In volume (mm³)	4819 ± 5523	9809 ± 14,145	13,215 ± 15,171	0.15
Wash-out ratio in per cent	31 ± 18	35 ± 20	10 ± 7	0.008
ADC decreased volume (mm³)	4432 ± 4808	4796 ± 6110	3573 ± 5194	0.88

Legend: mm³ – cubic millimeter, rCBV –relative cerebral blood volume, ADC – apparent diffusion coefficient

* - not calculated due to insufficient sample size,

** - descriptive data

The final diagnosis of tumor recurrence versus radiation necrosis was histopathologically confirmed in 20 patients (14 tumor recurrence, 4 radiation necrosis, 2 mixed pathologies) and based on longitudinal MRI follow-up in 13 patients (9 tumor recurrence, 2 radiation necrosis, 2 mixed pathologies). In two of the latter cases, FET-PET was additionally used to support the diagnostic classification.

### Histopathology

Molecular and immunohistochemical analyses of 25 patients with Gb revealed 13 cases with TERT C228T mutations and four cases with ATRX loss. MGMT promoter methylation was detected in 75.9% of evaluable patients with Gb or astrocytoma (22/29). The mean MIB-1/Ki-67 proliferation index was 23.1% (± 13.5%), and the mean p53 expression index was 34.0% (± 37.7%).

The patients with grade 4 astrocytoma had an IDH1-R132H mutation (both MGMT promoter-positive and without ATRX loss, MIB-1 30 and 40%, p53 10% and 80%).

Of the two grade 3 gliomas, one was identified as an IDH2-wildtype xanthoastrocytoma, and the other as an astrocytoma harboring MGMT promoter methylation and an IDH1 R132H mutation. The patient with primary CNS lymphoma histologically suffered from diffuse large B-cell Non-Hodgkin Lymphoma. Patients with brain metastases had cerebral metastatic melanoma (*n* = 2) and NSCLC (*n* = 1, adenocarcinoma).

### Time to second diagnosis

All data in this section refer to histologically confirmed cases only. The average time to second surgical intervention was 2.59 ± 2.76 years (mean ± standard deviation) for progressive disease, 0.72 ± 0.89 years for mixed pathology, and 2.95 ± 3.32 years for radiation necrosis, with median values of 1.48 (interval 0.40-10.42), 0.72 (interval 0.09–1.35), and 1.61 (interval 0.39–8.75) years, respectively. No cases of pseudoprogression were identified; all therapy-associated changes in our cohort represented radiation necrosis. Based on the timing of their occurrence, pseudoprogression cannot be excluded as a component of the mixed-pathology cases.

The time to PET (for cases without second surgical intervention) was 1.04 ± 0.14 years.

### MR Perfusion

**Fig. 3:**
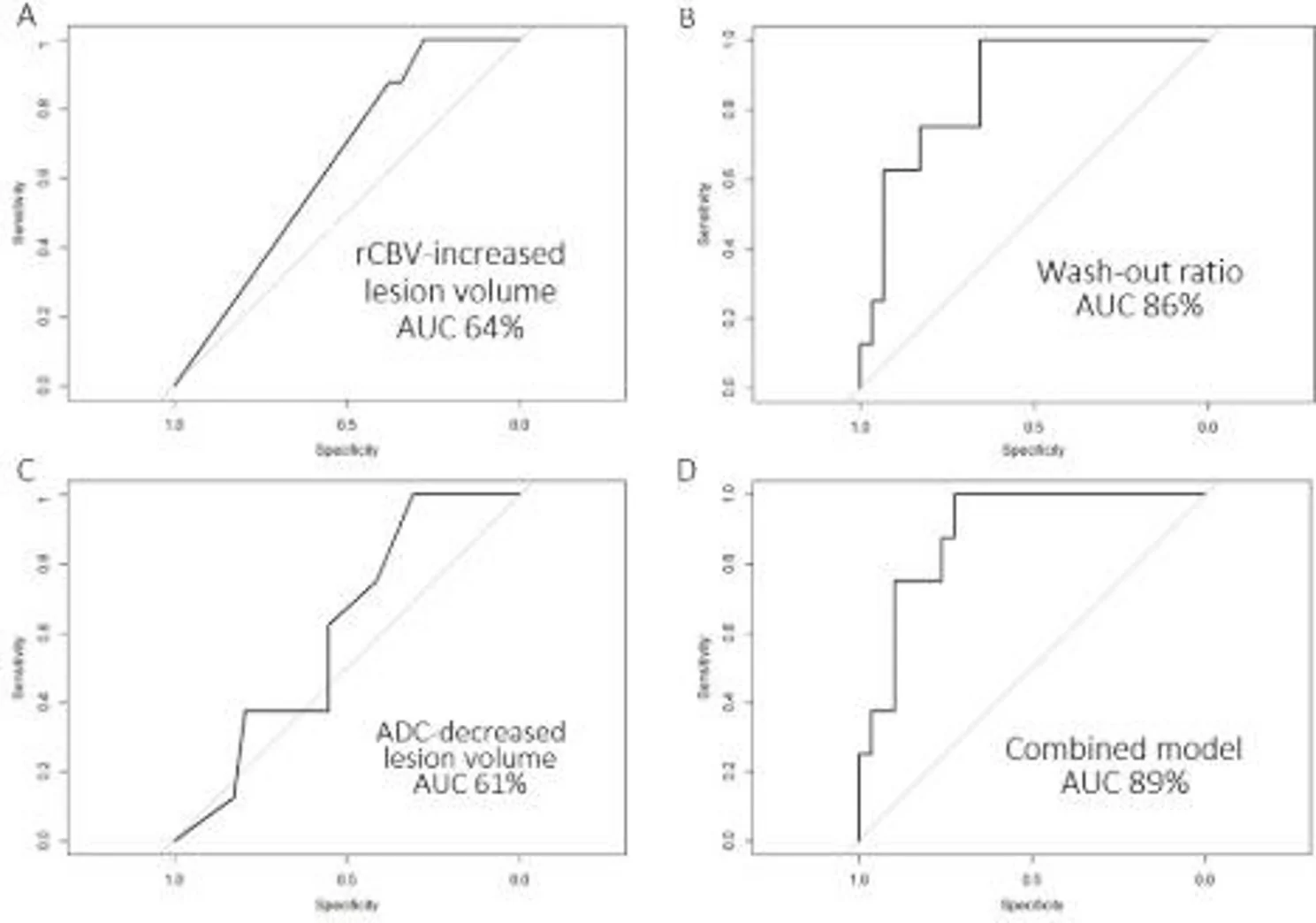
Area-under-Curve Receiver Operating Characteristics curves for the detection of radiation necrosis of (**A**) the hyperperfused lesion volume in percentage, (**B**) the washout ratio, (**C**) the ADC-lowered lesion volume in percentage, and (**D**) the combined probability multivariate model of rCBV-increased lesion volume, ADC-decreased lesion volume, and washout ratio

In 14 of 31 patients with MR perfusion, rCBV was significantly elevated (rCBV > 1.5) with an average volume of 46% ± 40% (mean ± standard deviation) of the suspected lesion. In 10 of 21 patients with tumor recurrence and available MR perfusion, hyperperfusion was found, with 53 ± 38% of the lesion volume hyperperfused. In three of four patients with mixed lesions (“radiation necrosis with scattered active tumor cells”), rCBV elevation was detected, with a mean hyperperfused lesion volume of 36 ± 55%. In one patient with histologically confirmed radiation necrosis, rCBV elevation was observed in 5% of the tumor volume. No measurable confluent hyperperfusion was detected in the other five patients with radiation necrosis and available MR perfusion data. The sensitivity of MR perfusion (as a true-false value) for the detection of radiation necrosis was 83% (5/6, CI 35,9–99,6%), with a low specificity of 52% (13/25). The AUC analysis for volume is shown in Fig. 3A with a threshold of ≤ 5% of hyperperfused volume with a sensitivity of 100% and specificity of 28%; the AUC was 0.64 (CI 0.42–0.84).

### Washout maps

The mean washout ratio for recurrent brain tumors (23 patients) was 31 ± 18% (Gb 36 ± 24%, *n* = 18, CNS lymphoma 60%, *n* = 1, brain metastasis 23 ± 19%, *n* = 2, astrocytoma grade 4 30 ± 10%, *n* = 2). Mixed pathologies in four patients with Gb revealed an average washout ratio of 35 ± 20%. In the six patients with radiation necrosis, the mean washout ratio was 10 ± 7% (Gb: 18 ± 3%, *n* = 3; astrocytoma grade 3: 5 ± 2%, *n* = 2; CNS lymphoma: none; brain metastasis: 9%, *n* = 1).

ROC analysis revealed an optimal exploratory threshold of < 23% for the washout ratio, with a sensitivity of 100% (95% CI, 54–100%) and a specificity of 67% (95% CI, 48–82%) for the detection of radiation necrosis. The washout ratio differentiated tumor progression from radiation necrosis with an AUC of 0.86 (95% CI, 0.75–0.99).

An alternative threshold of 15% demonstrated a sensitivity of 75% and a specificity of 85%. Figure 3B shows the ROC curve.

### Subgroup analysis for histologically confirmed diagnosis

The mean washout ratio for the histologically confirmed recurrent brain tumors (14 patients) was 34 ± 20% (Gb: 34 ± 22%, *n* = 9; astrocytoma grade 4: 31 ± 10%, *n* = 2; CNS lymphoma: 60%, *n* = 1; brain metastasis: 19 ± 23%, *n* = 2). In the four patients with histologically confirmed radiation necrosis, the mean washout ratio was 12 ± 7%.

At an exploratory threshold of < 24%, sensitivity was 100% (4/4; 95% CI, 41–100%) and specificity was 78.6% (11/14; 95% CI, 49–95%) for the detection of radiation necrosis. The washout ratio differentiated tumor progression from radiation necrosis with an AUC of 0.84 (95% CI, 0.67-1.00).

### ADC lowered lesion volumes

The mean lesion volume of decreased ADC values was 53 ± 41% for progressive disease, 39 ± 35% for mixed pathology, and 25 ± 33% for radiation necrosis. AUC analysis yielded two exploratory thresholds with an equivalent Youden index: <70% (sensitivity 86%, specificity 45%) and < 80% (sensitivity 100%, specificity 31%). Both operate at essentially the same combined sensitivity and specificity (Fig. 3C; AUC 0.61, CI 0.39–0.82).

### Multivariable analysis

Multivariable logistic regression was performed to assess the combined diagnostic values of the ADC, washout ratio, and perfusion parameters. The combined AUC was 0.89 for differentiating radiation necrosis from active tumor cells (tumor progression/mixed pathology). Figure 3D shows the ROC curve. Predicted probabilities derived from the model were used for ROC analysis, and the optimal threshold was a model probability of 0.16 for the detection of radiation necrosis, with a sensitivity of 100% and specificity of 72%.

### Interrater- and intra-rater reliability

Washout volume measurements demonstrated excellent interrater reliability, with an ICC(2,k) of 0.92 (95% confidence interval [CI]: 0.80–0.96). Reliability was lower for rCBV-derived hyperperfused and ADC-decreased volumes, with ICC(2,k) values of 0.79 (95% CI: 0.65–0.86) and 0.70 (95% CI: 0.39–0.85), respectively.

Intra-rater reliability was assessed in 15 cases by repeating the washout-volume segmentation after a four-week interval and showed excellent agreement (ICC, 0.99; 95% CI, 0.982–0.997).

## Discussion

In our retrospective cohort of 33 patients, the washout ratio differed significantly between radiation necrosis and tumor progression and was useful for differentiating these entities. An exploratory threshold of 23% was identified for the washout ratio. Perfusion and diffusion-weighted imaging appeared to provide additional diagnostic information but did not show the same discriminatory value in this cohort. Given the limited sample size and retrospective design, both the observed group difference and the identified threshold should be regarded as preliminary and hypothesis-generating rather than as evidence supporting a specific clinical imaging strategy or a generally applicable diagnostic cutoff.

Our group recently introduced washout maps as a faster contrast-kinetic approach [13, 24]. In the present cohort, the washout ratio showed a significant difference between tumor progression and radiation necrosis, and an exploratory threshold of 23% was identified for differentiating these entities. This observation supports further investigation of washout mapping as a potential imaging biomarker. However, because the threshold was derived from a small retrospective cohort with heterogeneous histologies and incomplete histopathological confirmation, it may be affected by overfitting and should not be regarded as a validated clinical cutoff. The present results therefore do not establish washout maps as a standalone diagnostic method or demonstrate superiority over established perfusion techniques.

Distinguishing true tumor progression from pseudoprogression and radiation necrosis remains a central challenge in post-treatment neuro-oncological imaging. On conventional MRI, all three entities may present with new or increasing contrast enhancement, surrounding edema, and mass effects, particularly after radiotherapy and temozolomide-based treatment. This overlap is clinically important because misclassification can lead to either premature discontinuation of effective therapy or delayed treatment of the true recurrence. Current response frameworks therefore emphasize caution: according to RANO 2.0 [5], for tumors treated with radiotherapy, post-radiotherapy MRI should be used as the baseline study, and imaging findings suggestive of progression within three months after completion of radiotherapy should generally be confirmed on follow-up imaging before being labeled as true progression [4].

Pseudoprogression is generally understood as a transient treatment-related increase in enhancement caused by therapy-induced vascular injury and blood-brain barrier disruption rather than viable tumor growth [6, 25]. It most often appears within approximately 3–4 months after radiotherapy, although the exact timing can vary. In contrast, radiation necrosis is usually considered a later treatment effect, typically developing 3–12 months after radiotherapy, but it may also occur years later [26]. These temporal patterns are helpful but not definitive because substantial overlap exists, especially in patients with aggressive diseases or complex treatment histories [6].

In this setting, MR perfusion has become one of the most widely used advanced imaging techniques because it interrogates tumor vascularity rather than relying only on lesion size or enhancement [27]. The biological rationale is that viable tumor progression is usually associated with neovascularity and increased microvascular density, which tend to produce higher perfusion metrics, especially rCBV, whereas pseudoprogression and radiation necrosis more often reflect endothelial injury, coagulative necrosis, and treatment-related inflammatory changes, which tend to show lower perfusion [28]. In the literature, DSC perfusion MRI has shown a useful diagnostic performance for this problem. A meta-analysis reported pooled sensitivity and specificity of 0.84 and 0.78, respectively, for DSC perfusion MRI in distinguishing tumor progression from treatment-related changes [29]. Another meta-analysis found pooled sensitivity and specificity of 0.82 and 0.87, respectively, for differentiating recurrence from pseudoprogression [30].

Among perfusion methods, DSC-MRI remains the most established in routine practice, largely because rCBV is familiar, fast to obtain, and supported by the largest evidence base. However, an important limitation of this approach is the lack of universal standardization of the data. Perfusion performance depends on preload dosing, pulse-sequence design, flip angle, leakage correction, post-processing software, and region-of-interest strategy [31]. This variability significantly affects interpretation. Studies have shown that quantitative interpretation in the post-treatment setting can be highly variable between software and readers, and recommend caution, including double reading, when DSC is used for recurrence surveillance [32]. Furthermore, more recent work suggests that volumetric or semi-automated analysis approaches may outperform simple hotspot measurements, underscoring that the methodology strongly influences diagnostic performance [33–35]. Artificial intelligence may further improve the evaluation of MR perfusion imaging and potentially enable the development of novel perfusion techniques [36, 37].

Treatment Response Assessment Maps (TRAMs), also known as contrast clearance analyses, offer a complementary approach [38]. TRAMs are generated from two post-contrast T1-weighted acquisitions obtained at different time points, typically including a delayed scan performed more than one hour after contrast administration [39]. This method is based on contrast kinetics rather than susceptibility effects. Areas of contrast washout or clearance are thought to correspond more often to viable tumors with hypervascularity and rapid contrast turnover, whereas areas of contrast accumulation are thought to represent treatment-related tissue injury, scarring or necrosis [38]. A histology-validated study demonstrated that regions of contrast clearance on TRAMs identified active tumors with 100% sensitivity and 92% positive predictive value, showing less susceptibility to hemorrhage or calcification artifacts than DSC-based methods [38].

The practical importance of TRAMs and washout maps lies in their ability to address several weaknesses of DSC perfusion. Because TRAMs rely on delayed T1 contrast behavior rather than T2* signal changes, they are less affected by susceptibility artifacts from blood products, postoperative material, air-bone interfaces, or calcification [40]. This can be particularly valuable in postoperative glioblastoma cavities or hemorrhagic lesions, where DSC maps may degrade.

TRAMs and washout maps are also visually intuitive, which may improve their clinical usability. Studies suggest that TRAMs can support the differentiation of recurrent malignant glioma from pseudoprogression and radiation necrosis, particularly when conventional MRI and perfusion findings are inconclusive [41, 42].

However, the evidence base for TRAMs remains more limited than that for MR perfusion. Although initial studies were promising, more recent investigations have highlighted limitations, such as small sample sizes and limited representation of radiation necrosis cases [41]. Therefore, TRAMs should be considered an adjunct rather than a replacement for established perfusion techniques.

Perfusion imaging provides a hemodynamic assessment of vascularity, whereas TRAMs characterize the delayed contrast kinetics within the same lesion. Concordant findings increase diagnostic confidence: high rCBV together with contrast washout on TRAMs supports tumor progression, whereas low perfusion combined with contrast accumulation favors pseudoprogression or radiation necrosis. Discordant findings should prompt careful multidisciplinary evaluation, short-interval follow-up, and histopathological confirmation when clinically required. This integrative approach aligns with modern response criteria, which emphasize the combination of imaging findings and the clinical context [7, 10].

In summary, MR perfusion, particularly DSC-derived rCBV, remains the most established and validated imaging biomarker for differentiating tumor progression from treatment-related effects in brain tumors. TRAMs and washout maps provide additional biologically meaningful information and may be especially useful in cases with susceptibility artifacts or inconclusive perfusion results. However, further large-scale validation and standardization are required before washout maps can be considered a standalone diagnostic tool. In clinical practice, the most reliable strategy remains a multiparametric approach integrating conventional MRI, treatment timing, clinical status, advanced perfusion imaging, washout maps, TRAMs when available, and, in selected cases, histopathology or additional modalities such as amino-acid PET.

Washout mapping and (FET-) PET assess different biological processes (delayed contrast kinetics and blood-brain barrier behavior versus amino acid transport and tumor metabolism, respectively) and could therefore provide complementary information. Direct comparative studies are needed to determine whether washout mapping can achieve diagnostic performance comparable to FET-PET or provide incremental value when both techniques are combined.

### Limitations

Several limitations should be acknowledged. First, the retrospective nature of the study may have resulted in selection bias and reduced control over confounding factors. Second, the cohort size was relatively small, which reduced statistical power and may have limited the generalizability of the findings. Third, the study population included patients with different histologies, potentially introducing biological heterogeneity that could influence the treatment response and outcome assessment. The small cohort and limited number of radiation-necrosis cases increase the risk of model overfitting; therefore, the combined model and ROC-derived thresholds should be considered exploratory and require validation in an independent cohort. Finally, diagnosis and response evaluation were partly based on follow-up and PET-controlled assessments rather than uniform histopathological confirmation in all cases, which may have affected the diagnostic consistency.

A single rCBV threshold of > 1.5 was used to define elevated perfusion despite the inclusion of data from different scanners and field strengths; therefore, scanner- and post-processing-related variability may have affected rCBV classification and limits the generalizability of this threshold.

## Conclusion

Washout and late enhancement provide additional diagnostic information and may improve lesion characterization in Gb. Therefore, a combined approach integrating washout, late enhancement, ADC values, and perfusion imaging seems to be a promising strategy for future diagnostic assessments.

The technique is applicable to various types of malignant brain tumors; however, its broader applicability, validity, and generalizability remain to be determined in larger and independent cohorts.

## Supplementary Information

Below is the link to the electronic supplementary material.

Supplementary Material 1

## Data Availability

The datasets used and/or analyzed during the current study are available from the corresponding author upon reasonable request.

## Abbreviations

ADC: Apparent Diffusion Coefficient
AI: Artificial Intelligence
ASL: Arterial Spin Labeling (MR perfusion)
AUC: Area under Curve
CI: confidence interval
DSC: Dynamic Susceptibility Contrast (MR perfusion)
DWI: Diffusion-Weighted Imaging
EPI-DWI: echo-planar diffusion-weighted imaging
FET-PET: ^18^F-fluoroethyl-L-tyrosine positron emission tomography
FLAIR: fluid-attenuated inversion recovery
FLIRT: FMRIB's Linear Image Registration Tool
FMRIB: Functional MRI of the Brain
ICC: intraclass correlation coefficient
IDH: isocitrate dehydrogenase
IQR: interquartile range
MGMT: O6-methylguanine-DNA methyltransferase
PACS: Picture Archiving and Communication System
PET: Positron Emission Tomography
rCBV: relative Cerebral Blood Volume
ROC: Receiver Operating Characteristic
SD: Standard Deviation
TERT: telomerase reverse transcriptase
TRAMs: Treatment Response Assessment Maps
