# Supplementary material for: Late enhancement and washout maps for distinguishing tumor progression from radiation necrosis in malignant brain tumors: an exploratory study
Source: BMC Cancer. 2026 Oct 15;26:1238. doi: 10.1186/s12885-026-17170-0 (PMC13673850; doi:10.1186/s12885-026-17170-0)
Supplement: Supplementary file 1 [file 12885_2026_17170_MOESM1_ESM.docx]

**SUPPLEMENTARY MATERIAL**

**Table S1.** Basic data of the included patients.

| **Variable** | **Overall** | **Gb** | **Astrocytoma WHO grade 4** | **Astrocytoma WHO grade 3** | **CNS lymphoma** | **Brain metastasis** |
| --- | --- | --- | --- | --- | --- | --- |
| n | 33 | 25 | 2 | 2 | 1 | 3 |
| Age at diagnosis (years) | 58.8 ± 12.2 | 62.5 ± 7.7 | 60.0 ± 26.8 | 30.5 ± 9.4 | 49.0 ± 0.0 | 50.3 ± 10.9 |
| Age at new contrast enhancing lesion (years) | 61.1 ± 11.4 | 64.0 ± 7.7 | 64.0 ± 24.1 | 36.0 ± 14.1 | 59.0 ± 0.0 | 53.3 ± 10.8 |
| Females in % | 39 | 40 | 50 | 50 | 0 | 33 |

Legend: Gb – glioblastoma; WHO –World Health Organization; CNS – central nervous system

**Description D1.** Calculation of wash-out maps.

Wash-out maps were generated using an in-house developed pipeline implemented in Python 3.11. Initially, both MRI series were converted from DICOM (Digital Imaging and Communications in Medicine) to NIfTI (Neuroimaging Informatics Technology Initiative) format using the dicom2nifti library (version 2.4.10). Subsequently, N4 bias field correction was performed using SimpleITK (version 2.3.1), with a threefold reduction applied during the correction process. To ensure comparability between the two MRI series, both datasets were then intensity-normalized to an identical mean signal value. The normalized images were subsequently aligned using rigid image registration with FLIRT (FMRIB’s Linear Image Registration Tool) provided by the FMRIB Software Library (FSL, version 6.0).

Following registration, the second MRI series was subtracted from the first on a voxel-by-voxel basis. The resulting subtraction maps were converted back into DICOM format and exported as both grayscale and RGB (Red, Green, Blue) image series. For visualization of the RGB maps, the intensity range was initially reduced by a factor of two and subsequently centered around a value of 127 on the 8-bit intensity scale (0–255). Negative subtraction values were represented in green, whereas positive values were displayed in red. Accordingly, regions with higher signal intensity in the first MRI series relative to the second appeared red, while regions with lower signal intensity appeared green.
